# Control of Tongue Position in Patients with Obstructive Sleep Apnea: Concept and Protocol for a Randomized Controlled Crossover Trial

**DOI:** 10.3390/ijerph20116026

**Published:** 2023-06-01

**Authors:** Tatsuya Fukuda, Yuuya Kohzuka, Fernanda R. Almeida, Takehiko Iijima, Rikuo Masuda, Satoru Tsuiki

**Affiliations:** 1 Research Department, Institute of Neuropsychiatry, 91, Bentencho, Shinjuku-ku, Tokyo 162-0851, Japan; tacchan.69@icloud.com; 2 Department of Perioperative Medicine, Division of Anesthesiology, Showa University School of Dentistry, 2-1-1 Kitasenzoku, Ota-ku, Tokyo 145-8515, Japan; kohyu@dent.showa-u.ac.jp (Y.K.); iijima@dent.showa-u.ac.jp (T.I.); rikuo14@dent.showa-u.ac.jp (R.M.); 3 Department of Dental Anesthesiology, Showa University Koto Toyosu Hospital, 5-1-38 Toyosu, Koto-ku, Tokyo 135-8577, Japan; 4 Department of Oral Health Sciences, Faculty of Dentistry, The University of British Columbia, 2199 Wesbrook Mall, Vancouver, BC V6T 1Z3, Canada; falmeida@dentistry.ubc.ca; 5 Division of Aging and Geriatric Dentistry, Department of Oral Function and Morphology, Tohoku University Graduate School of Dentistry, 4-1 Seiryo-Machi, Aoba-ku, Sendai 980-8575, Miyagi, Japan

**Keywords:** obstructive sleep apnea, randomized controlled trial, intravenous sedation, tongue position retainer

## Abstract

We hypothesize that the control of tongue position using a newly developed tongue position retainer, where the tongue is held in a protruded position (i.e., intervention A) or in its resting position (i.e., intervention B), is effective for maintaining upper airway patency in obstructive sleep apnea (OSA) compared with no control of tongue position. This is a randomized, controlled, non-blinded, crossover, and two-armed trial (i.e., sequence AB/BA) in 26 male participants (i.e., sample size) who are scheduled to undergo a dental operation under intravenous sedation with OSA (10 ≤ respiratory event index < 30/h). Participants will be randomly allocated into either sequence by a permuted block method, stratified by body mass index. Under intravenous sedation, participants will undergo two interventions, separated by a washout period after receiving intervention A or intervention B using a tongue position retainer after baseline evaluation, before each intervention is provided. The primary outcome is the abnormal breathing index of apnea as determined by the frequency of apnea per hour. We expect that, compared with no control of tongue position, both intervention A and intervention B will improve the abnormal breathing events with superior effects achieved by the former, offering a therapeutic option for OSA.

## 1. Introduction

The excitability of hypoglossal motoneurons innervating the genioglossus muscle, the main tongue protruder, is markedly suppressed during sleep in both healthy subjects and patients with obstructive sleep apnea (OSA) [1]. However, during wakefulness, apneic events hardly ever occur, regardless of the presence or absence of such episodes during sleep, because the genioglossus muscle reflectively augments its activity as needed to maintain the tongue in position to protect the upper airway patency [2]. Based on this phenomenon, we hypothesized that OSA could be alleviated if the resting tongue position during wakefulness can be maintained during sleep independently of genioglossus muscle activity [3].

**Figure 1:**
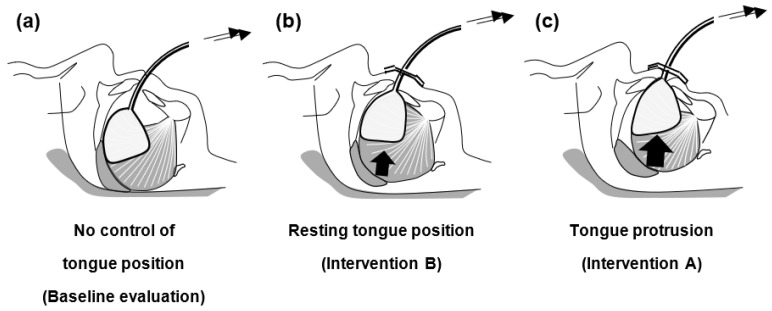
Schematic representation of the possible effects of control of tongue position on upper airway patency in obstructive sleep apnea (OSA): a hypothesis that apneic events occur due to a reduction in genioglossus muscle activity during sleep/intravenous sedation. Although the tongue position retainer (TPR) will lead to tongue collapse if subjects solely wear the device (no control of tongue position) (**a**), tongue collapse could be alleviated by holding the tongue using TPR in its resting position (intervention B) (**b**) and in a protruded position (intervention A) (**c**), with superior effects being achieved by intervention A (**c**). Note that no control of tongue position (**a**) and resting tongue position (**b**) are different maneuvers.

In exploratory studies, we demonstrated that the maintenance of resting tongue position by continuous tongue suction improved OSA [3,4]. However, in subsequent trials, we also experienced treatment failure with a larger tongue; although the tongue appeared to be held throughout the night by tongue suction, the device likely allowed for the tongue base to collapse. This could indicate that not only maintaining resting tongue position per se, but also additional tongue protrusion, might mitigate tongue collapse more efficiently in OSA patients, who generally have larger tongues in comparison with non-OSA people [5,6,7,8]. Accordingly, the objective of this study is to test the following hypothesis (Figure 1); Compared with no control of tongue position, holding the tongue in a protruded position and in its resting position will improve the abnormal breathing events, with superior effects being achieved by the former in OSA patients. We designed a randomized controlled trial (RCT) to investigate the effects of controlling tongue position using a newly developed tongue position retainer (TPR) on upper airway patency in OSA during intravenous sedation. Our concept may lead to the development of different approaches to continuous positive airway pressure (CPAP) and mandibular advancement devices for OSA.

## 2. Materials and Methods

### 2.1. Study Design

**Figure 2:**
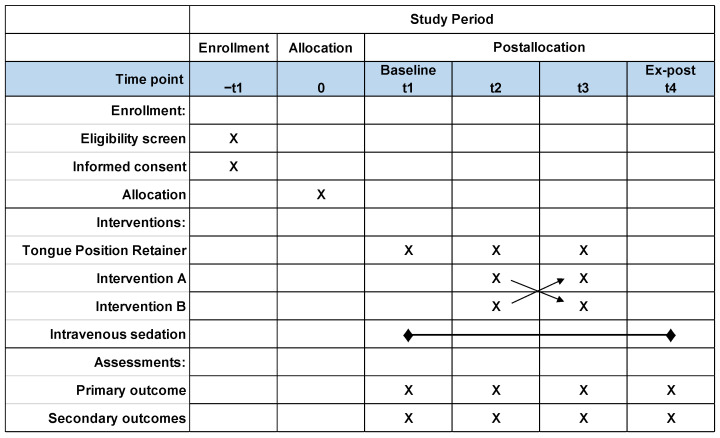
Study overview and Standard Protocol Items: Recommendations for the Interventional Trials (SPIRIT). Interventions and assessments will be administered at different time points indicated by X.

This is a randomized, controlled, non-blinded, crossover, and two-armed (i.e., sequence AB/BA) clinical trial that will follow the CONSORT 2010 statement, providing an extension to randomized crossover trials to better report the results [9]. An overview of the study is presented in Figure 2, following the recommended template for the schedule of enrollment, interventions, and assessments described by Standard Protocol Items: Recommendation for Interventional Trials (SPIRIT) [10]. Recruitment will last from the day the data are released by the Japan Registry of Clinical Trials (jRCTs) (032220040) (25 April 2022), and the duration of the study itself will start from the day the data are released by jRCTs until 31 March 2026.

### 2.2. Ethical Considerations

The protocol for the present study was submitted to and approved by the institutional ethics committee at the Showa University Clinical Research Review Board, Tokyo, Japan (protocol version 1.1, approval no. S11). After this approval, the study was registered as an RCT in jRCTs on 25 April 2022, which was further amended on 26 December 2022 (protocol version 1.3). Full details of all protocol amendments are provided within the protocol (https://jrct.niph.go.jp/en-latest-detail/jRCTs032220040: accessed on 28 May 2023). All the participants will be informed of the underlying principles, the purpose of the trial with respect to the intervention, and possible slight discomfort of the tongue capsule and will provide their written consent before the initiation of the study. In addition, this study will be conducted in accordance with both the amended Declaration of Helsinki and the Clinical Trials Act [11].

### 2.3. Participants

#### 2.3.1. Inclusion Criteria

(1) Patients who are scheduled to undergo a dental operation under intravenous sedation at Showa University Koto Toyosu Hospital.
(2) Males aged > 20 years.
(3) Patients diagnosed with OSA [10/h ≤ respiratory event index (REI) < 30/h] based on out of center sleep testing (OCST; also referred to as home sleep apnea testing or type III portable monitoring) (PMP-300E, Pacific Medico Co., Ltd., Tokyo, Japan) [12,13,14,15]. Assessment will be performed by registered polysomnographic technologists in accordance with the American Academy of Sleep Medicine Scoring Manual [16].
(4) Patients who are healthy, or who have mild systemic disease in accordance with American Society of Anesthesiologists physical status I or II [17].
(5) Patients who agree to participate in this study and provide their written consent to participate in the study.

#### 2.3.2. Exclusion Criteria

(1) Patients with cognitive dysfunction and/or sensor impairment.
(2) Subjects assumed to be ineligible by the principal investigator or co-investigators.
(3) Patients who cannot provide their written consent.
(4) Patients in whom the tongue cannot be maintained in position by the TPR.

### 2.4. Intervention

**Figure 3:**
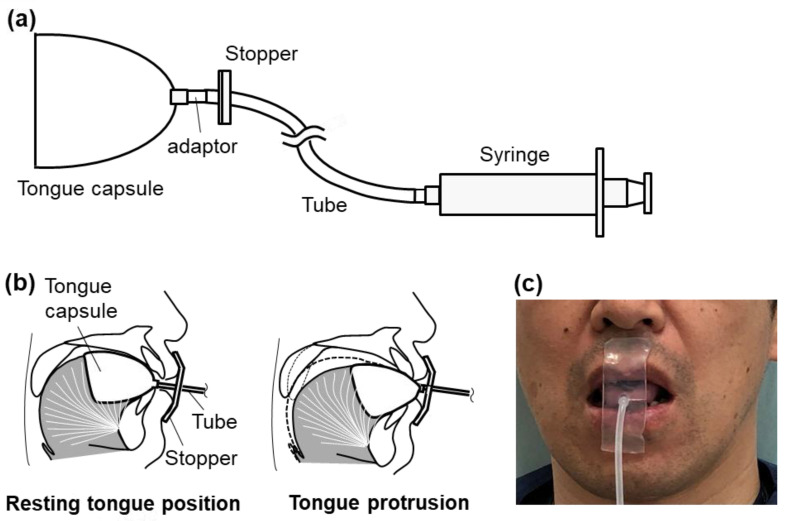
Tongue position retainer (TPR) for the treatment of obstructive sleep apnea. Top view of the TPR, which consists of five parts: tongue capsule, silicone tube, adaptor, stopper, and syringe (**a**). Lateral schematic illustration of the TPR in place with resting tongue position ((**b**) left) and with tongue protrusion ((**b**) right). Frontal view of the TPR in place (**c**).

Control of tongue position during intravenous sedation will be performed using a TPR. The TPR consists of five parts: (1) tongue capsule (SK Corporation Ltd., Tokyo, Japan), (2) tube made of silicone (SK Corporation Ltd., Tokyo, Japan) (3) adapter made of polyacetal that connects the tongue capsule to the tube, (4) vacuum syringe to generate negative pressure (10 mL, VACKLOK, Merit Medical Japan, Tokyo, Japan), and (5) stopper situated in front of the upper and lower lips to hold the tongue capsule (CGK, Tokyo, Japan) (Figure 3). Once the tongue is sucked by the light negative pressure produced by the vacuum syringe, the plunger design of the syringe should maintain the negative pressure with ease and will not inadvertently disengage. The stopper is firmly placed on the silicone tube, which prevents dorsal displacement of the tongue capsule via the tube. The tongue position is maintained as long as the tongue does not fall from the capsule. Since the investigator can position the stopper at a freely selected location on the tube, the tongue will be advanced by decreasing the distance between the stopper and the tongue capsule. In addition, the amount of tongue protrusion can be easily calibrated as a change in the distance between the stopper and the tip of the tongue capsule.

TPR will allow for tongue collapse if subjects solely wear the TPR (Figure 1a). On the other hand, resting tongue position maneuver with TPR is assumed for intervention B, and instead the tongue is held with the TPR at its resting position, where the tip of the tongue capsule is situated at the edge of the mandibular incisors (MAX 0) (Figure 1b). The tongue protrusion maneuver for intervention A is defined as holding the tongue without any soreness or discomfort using the TPR at 50% of the maximum tongue-advanced position (MAX 50) following holding the tongue using the TPR at the maximum tongue-advanced position (MAX 100) (Figure 1c).

### 2.5. Sample Size and Allocation Concealment Mechanism

In a previous study that evaluated the effect of tongue protrusion using a tongue-stabilizing device on the severity of OSA, Apnea Hypopnea Index (AHI) improved from 21.8 ± 8.6/h to 9.3 ± 5.8/h [18]. This indicates that, if intervention B is applied to a participant, there should be no change in AHI. Therefore, our null hypothesis is that the mean AHI was not different between intervention A (μ1) and intervention B (μ2) (i.e., μ1 = μ2), whereas the alternative hypothesis is that the mean AHI of intervention B is greater than that of intervention A (i.e., μ1 < μ2). The effect size (Δ0) and the power (i.e., 1-β) should be calculated with the following formula: Δ0 = (μ1 − μ2)/σ, 1 − β = Pr{t0 < −t(φ, 2α)}, φ = n1 + n2 − 2, where σ is the population standard deviation, β is the Type II error, φ is the degree of freedom, α is the Type I error set at 0.05, n1 is the number of subjects with intervention A, and n2 is the number of subjects with intervention B. Statistically, n1 and n2 are assumed to be equal (i.e., n1 = n2) to each other and 1-β is set at 0.80. Consequently, each intervention group needs 9 participants (i.e., 18 participants for both groups). If the drop-out rate is set rather high, at 30%, the study needs 25.7 samples; thus, a total of 26 participants will be required to test the hypothesis.

These 26 participants are to be randomized by creating a computer-generated randomization list using the permuted block method (Mujinwari, https://mujinwari.biz, Iruka-system, Tokyo, Japan: accessed on 28 May 2023) with random block sizes of two or four, stratified by body mass index (<25 kg/m^2^ or 25 kg/m^2^≤). Consequently, participants will be assigned random numbers based on consecutive enrollment into either sequence AB or sequence BA, which provides an approximately one-to-one ratio for the number of subjects in the two sequences.

### 2.6. Study Protocol

Eligible patients will be referred to the Department of Dental Anesthesiology, Showa University, Koto Toyosu Hospital to provide their written informed consent for the present study after an approximately 30 min detailed explanation of the purpose, schedule, risks, merits/demerits, and protocols of the present study by one of the investigators (Y.K.). This explanation will be associated with a test of the tongue capsule for each candidate. If the tongue capsule does not fit the subject’s tongue or causes excessive irritation/discomfort/soreness, etc., the participant will be excluded from the study. All the candidates will be scheduled for an evaluation of OSA using OCST, as described before. Subjects who are diagnosed with OSA with 10 ≤ REI < 30/h will be scheduled for the RCT, while those with REI < 10/h and 30/h ≤ REI will be excluded from the study at this stage.

**Figure 4:**
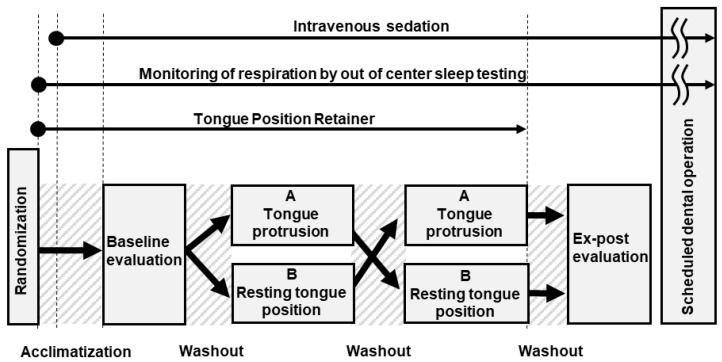
Schematic diagram summarizing the study protocol on the day of study. Monitoring of physiological parameters using out-of-center sleep testing as well as intravenous sedation will continue until the scheduled dental operation ends. See Materials and Methods Section for more details.

Prior to the induction of intravenous sedation on the study day, electrodes for OCST (LS-300, Fukuda Denshi Co., Ltd., Tokyo, Japan) will be attached to a patient sitting on a reclining dental chair to allow for the monitoring of nasal pressure via a nasal cannula to assess nasal airflow, chest wall movements for assessing respiratory efforts, and arterial oxygen saturation (SpO_2_) using pulse oximetry (Figure 4). A routine vital-sign monitor including electrocardiogram, noninvasive blood pressure and an electroencephalogram (Root + SedLine, Masimo Corp., CA, USA) will be attached. Thereafter, one of the investigators (Y.K.) and the subject will simulate the study with the TPR in place while awake so that it can be smoothly performed under intravenous sedation.

The predetermined random allocation order will be strictly applied to all consecutive consenting patients during the study period. The anesthesiologist (Y.K.) will be informed of the allocation of the participant immediately before the initiation of intravenous sedation to reduce potential bias. The anesthesiologist will provide conscious intravenous sedation by a bolus intravenous injection of midazolam (1 to 3 mg) and propofol (5 to 10 mg), followed by continuous infusions of propofol (1 to 3 mg kg^−1^ h^−1^) using the target-controlled infusion system [19,20]. After an acclimatization period for TPR (30 s), a baseline evaluation (i.e., no control of tongue position with TPR in place) will be performed (1 min) (Figure 4). Thereafter, each patient will undergo both interventions A (MAX50 for 1 min and MAX 100 for 1 min) and B (1 min) in a randomized order with a washout period (30 s) between the interventions. An ex-post evaluation (1 min) will also be scheduled after removal of the TPR, when both study sequences are complete. After the core part of the present study (i.e., baseline evaluation, interventions, and ex-post evaluation) which will take an average of 6 min, supplemental oxygen at a flow rate of 3 L/min will be provided for the scheduled dental operation.

### 2.7. Study Outcome

#### 2.7.1. Primary Outcome

Since this study will be performed under intravenous sedation, and not during sleep, definitions of apnea and hypopnea as alternatives to AHI and REI that occur during sleep need to be prepared for scoring abnormal breathing. In other words, definitions for an intravenous sedation study as an alternative to AHI and REI and other variables for studies during sleep will more reasonably reflect the effects of the intervention on upper airway patency during intravenous sedation, as described in our previous report [20]. Abnormal breathing under intravenous sedation is to be predetermined by OCST based on five expected features of abnormal breathing: (1) obstructive or central, (2) apnea or hypopnea, (3) with or without desaturation, (4) with or without irregular breathing, and (5) bradypnea or tachypnea. Upper airway obstruction is determined by the presence of respiratory effort for an apnea, and the presence of inspiratory flow limitation is evidenced by a flattened nasal pressure signal for a hypopnea. Apnea, expressed as “A”, under intravenous sedation is defined by complete cessation of the nasal pressure changes for more than 10 s, whereas hypopnea, expressed as “H”, under intravenous sedation, is defined as being one of three types: H70, H50 and H30. H70 is defined as a more than 70% reduction in the nasal pressure amplitude for more than 10 s. H50 is defined as a more than 50% reduction in the nasal pressure amplitude for more than 10 s. H30 is defined as a more than 30% reduction in the nasal pressure amplitude for more than 10 s. Desaturation, expressed as “D”, is defined as a more than 2% reduction in SpO_2_ for more than 10 s. Irregular breathing, expressed as “I”, is determined when the nasal pressure signal frequency (respiratory rate) and amplitude fluctuate more than twofold after apnea or hypopnea. Irregular breathing is considered to be elicited by laryngeal stimulation with water inside the oral cavity and subsequent cough reflexes. The abnormal breathing index (ABI) is calculated as the number of these abnormal breathing instances per hour of the study period. The abnormal respiratory time (ART) is the duration of one abnormal breathing and is calculated as a mean and a percentage during study period. Similarly, the ABI of apnea (ABI-A) will be used as the primary outcome for a statistical analysis of the study hypotheses.

#### 2.7.2. Secondary Outcome

The ABI of hypopneas (ABI-H70, ABI-H50, ABI-H30), the ABI of desaturation (ABI-D), the ABI of irregular breathing (ABI-I), the mean of ARTs (ART-A ART-H70, ART-50, ART-H30, ART-D, ART-I), and the percentage of ARTs will be used as the secondary outcome for the statistical analysis [20]. The record of OCST, vital signs, electroencephalogram, anesthesia, medical records, questionnaires, and drop-out rate will also be used [21].

### 2.8. Statistical Analysis

The baseline measures will be compared with the measures obtained under interventions A and B. The normality of data will be tested by the Shapiro–Wilk test. Repeated-measures ANOVA with the Bonferroni correction for multiple testing, followed by Tukey’s honestly significant difference tests will be used as the parametric test for normally distributed data. If the data do not follow a normal distribution, a non-parametric test, the Kruskal–Wallis test with the Bonferroni correction for multiple testing followed by Dunn–Bonferroni tests, will be used. The data will be presented as either the mean ± standard deviation or median (interquartile range) for normal and non-normal distributions. All statistical analyses will be performed using SPSS software (version 25, IBM Japan, Tokyo, Japan). All *p* values are two-tailed and a *p* value of <0.05 is considered to indicate statistical significance.

## 3. Discussion

In our former exploratory studies using the prototype device, we obtained evidence that partly supports our hypothesis that the maintenance of tongue position by continuous tongue suction improved OSA [3,4]. This study will, in turn, support and extend the above concept of direct approach to the tongue for OSA treatment using a more sophisticated study design.

The present study and other similar studies have significant limitations, which will certainly provide challenges to overcome and opportunities to polish the study design. The participants will be patients who are originally scheduled to undergo a dental operation under intravenous sedation as soon as the study ends, indicating that the number of expected OSA samples will be limited. Moreover, we will use OCST for the evaluation of outcomes, since it is not feasible for us to perform polysomnography under the perioperative circumstances in the dental operation room. Patients whose baseline REI is <10/h will not be recruited, because our previous study demonstrated that these individuals will easily achieve the variety criteria for treatment success (e.g., more than 50% reduction in baseline AHI and/or follow-up AHI < 5/h with more than 50% reduction in baseline AHI) in oral appliance therapy [13]. Additionally, subjects with 30/h ≤ baseline REI (i.e., severe OSA) will not be included to minimize the confounding effects on primary and secondary outcomes. Conversely, an RCT under intravenous sedation is a meritorious study design to obtain outcomes with a higher evidence level within a limited time, as with our previous study [20]. Although the administration of propofol for intravenous sedation was reported to suppress REM sleep, and a greater suppression of upper airway muscle activity than in natural sleep can also occur, the sedation level can be carefully targeted to a certain level of sleep (e.g., stage II non-REM sleep) throughout the study as long as this is under the control of a skilled anesthesiologist with the target-controlled infusion system [19,20,22,23]. Additionally, the identification of the site of occlusion during intravenous sedation/sleep and site of action of TPR is also an important concern in future studies in order to increase the success rate of TPR treatment. In the human pharynx, three segments are present: the velopharynx (from the level of the hard palate to the free margin of the soft palate), oropharynx (from the free margin of the soft palate to the tip of the epiglottis), and hypopharynx (from the tip of the epiglottis to the vocal cords) [24,25]. The most common site of occlusion is the velopharynx, although upper airway narrowing also occurs in the oropharynx in OSA [24,26]. Notably, Kuna and Remmers stated there is no simple occlusion at the oropharynx [24]. The authors support these previous reports with regard to the site of occlusion because such conclusions were drawn by direct observation of the upper airway narrowing either at the passive or asleep pharynx using endoscopes, whereas a recent report demonstrated that the tongue-based obstruction of upper airway was successfully identified using ultrasound and magnetic resonance imaging [24,26,27]. Finally, a similar RCT protocol to the present study needs be designed for female OSA patients in future.

A great advantage of the TPR compared to CPAP and mandibular advancement devices is that the TPR does not require either positive airway pressure or mandibular advancement. Thus, TPR can avoid the adverse effects associated with the use of CPAP (e.g., nasal congestion, nosebleeds, claustrophobia, difficulties falling asleep, discomfort due to the interface, dryness, and skin and eye discomfort) and/or mandibular advancement devices (e.g., hypersalivation or decreased saliva production, pain and discomfort in the teeth and gums, occlusal abnormalities when waking up, and discomfort in the jaw muscles and temporomandibular joint) and can be used in patients who are contraindicated for CPAP and mandibular/tongue advancement devices [28]. Moreover, another prominent merit of the TPR is that it allows for jaw movement and oral breathing. The TPR is similar to tongue-retaining devices, including the tongue-stabilizing device, in that it directly approaches the tongue for OSA treatment. We believe that maintaining the tongue in position using the TPR (Figure 1b) will have a positive impact on upper airway patency compared with no control of tongue position (Figure 1a) because it alleviates tongue collapse due to the reduction in genioglossus activity. The tongue-retaining device does not have a similar effect to TPR because it is designed to hold the tongue solely in a protruded position [29]. In addition, the TPR may be more clinically practical than our prototype device for various reasons. For example, the tongue capsule made of a thin silicone material is designed to closely fit the ventrolateral surface of the tongue (Figure 3). Therefore, once the tongue is held in the tongue capsule, it does not need continuous active suction and can be maintained by the light negative pressure produced by the vacuum syringe. These modifications will help avoid problems associated with our prototype device as well as oral pressure therapy, such as dry mouth, noise due to the accumulation of saliva in the suction tube and the vacuum generator, the difficulty of applying suction pressure, and cumbersome device management, all of which would be important factors for improving long-term acceptance/adherence [3,4,30].

## 4. Conclusions

In conclusion, holding the tongue in a protruded position and in its resting position will improve the abnormal breathing events with superior effects by the former during intravenous sedation in OSA patients. The results will contribute to the design of a future RCT using polysomnography during sleep to obtain more clinically practical outcomes for the different OSA treatment to CPAP and mandibular advancement devices.

## 5. Patents

The tongue position retainer is patent-pending (PCT/JP/2022/41919; Japanese Patent Application No. 2021-125898).

## Data Availability

The datasets generated and/or analysed during the current study are available from the corresponding author upon reasonable request.
